# Phylogenetics Study to Compare Chloroplast Genomes in Four Magnoliaceae Species

**DOI:** 10.3390/cimb45110578

**Published:** 2023-11-16

**Authors:** Jianyun Zhao, Hu Chen, Gaiping Li, Maimaiti Aisha Jumaturti, Xiaomin Yao, Ying Hu

**Affiliations:** 1 Key Laboratory of National Forestry and Grassland Administration on Cultivation of Fast-Growing Timber in Central South China, College of Forestry, Guangxi University, Nanning 530004, China; jianyunzhao1997@163.com (J.Z.); l15383644628@163.com (G.L.); 13072922873@163.com (M.A.J.); yaoxiaominwork@163.com (X.Y.); 2 Guangxi Key Laboratory of Forest Ecology and Conservation, College of Forestry, Guangxi University, Nanning 530004, China; 3 Guangxi Key Laboratory of Superior Timber Trees Resource Cultivation, Guangxi Forestry Research Institute, Nanning 530002, China; chenhubeijing-2008@163.com

**Keywords:** Magnoliaceae, chloroplast genome, phylogenetic analysis

## Abstract

Magnoliaceae, a family of perennial woody plants, contains several endangered species whose taxonomic status remains ambiguous. The study of chloroplast genome information can help in the protection of Magnoliaceae plants and confirmation of their phylogenetic relationships. In this study, the chloroplast genomes were sequenced, assembled, and annotated in *Woonyoungia septentrionalis* and three Michelia species (*Michelia champaca*, *Michelia figo*, and *Michelia macclurei*). Comparative analyses of genomic characteristics, repetitive sequences, and sequence differences were performed among the four Magnoliaceae plants, and phylogenetic relationships were constructed with twenty different magnolia species. The length of the chloroplast genomes varied among the four studied species ranging from 159,838 bp (*Woonyoungia septentrionalis*) to 160,127 bp (*Michelia macclurei*). Four distinct hotspot regions were identified based on nucleotide polymorphism analysis. They were *petA-psbJ*, *psbJ-psbE*, *ndhD-ndhE*, and *rps15-ycf1*. These gene fragments may be developed and utilized as new molecular marker primers. By using *Liriodendron tulipifera* and *Liriodendron chinense* as outgroups reference, a phylogenetic tree of the four Magnoliaceae species and eighteen other Magnoliaceae species was constructed with the method of Shared Coding Sequences (*CDS*). Results showed that the endangered species, *W. septentrionalis,* is relatively genetically distinct from the other three species, indicating the different phylogenetic processes among Magnoliaceae plants. Therefore, further genetic information is required to determine the relationships within Magnoliaceae. Overall, complete chloroplast genome sequences for four Magnoliaceae species reported in this paper have shed more light on phylogenetic relationships within the botanical group.

## 1. Introduction

Chloroplasts of higher plants have an independent double-stranded chloroplast genome, usually 45 µm in length. In most plant species, the chloroplast genome generally contains two inverted repeats (*IRs*), *IRA* and *IRB*, which divide the entire chloroplast genome into four parts, with the rest of the chloroplast genome consisting of a large single-copy region (*LSC*) and a small single-copy region (*SSC*) [1]. Chloroplast is an organelle of endosymbiosis origin, and the chloroplast genome plays an important role in the molecular systematics analysis of plants, species identification, and breeding research [2]. The chloroplast genome sequences also show some differences in sequence and structure within plant species, which are reflected in changes in genome length, sequence, genes, etc. [3,4]. These differences can be more directly demonstrated through various analyses. In order to more clearly reflect the evolutionary relationship of these four Magnoliaceae plants and distinguish the differences between them, general scholars adopted the method of building phylogenetic trees [5]. Currently, there are three alternative approaches to phylogenetic studies based on the information contained in the chloroplast genome. The first approach utilizes the whole chloroplast genome sequence (*WCGS*), the second strategy is based on coding sequences (*CDS*) only, whereas the third method uses solely *matK* gene sequences [6,7]. The confidence level of *WCGS* and *CDS* is higher than that of *matK* gene sequences [6]. The evolutionary relationships revealed by *CDS* are closer to the real classification of species, and the results of phylogenetic trees constructed by *CDS* and *WCGS* are often more consistent [8]. The experimental material of this study is the cp genome, whose sequence length is shorter than that of nuclear gene fragments, while the matk gene sequence is a research method for short fragment sequences. Therefore, the CDS method will be adopted in this study to explore the phylogenetic relationship of magnolia species.

Magnoliaceae plants are dicotyledonous plants that are precious materials for studying the origin, development, and evolution of angiosperms. As one of the most primitive families of angiosperms, Magnoliaceae first manifests itself in morphology as pedicels columnar, with pistils separated, stamens spirally arranged, no distinct sepal-petal differentiation, and flowers are numerous and indefinite, etc.; second, the number of examples is scarce and some magnolia plants are on the verge of extinction [3]. The 18 genera and approximately 335 species in Magnoliaceae are mainly distributed in southeastern and southern Asia, southeastern North America, and northern South America [9]. There are 14 genera and approximately 165 species of Magnoliaceae in China, mainly distributed in southeastern to southwestern China. In April 2022, there were approximately 80 plastid genomes of Magnoliaceae species registered in the National Center for Biotechnology Information (NCBI; accessed on 2 April 2022; https://www.ncbi.nlm.nih.gov/), including the representatives of the following genera of Magnolia, Liriodendron, and Kmeria. In Magnoliaceae, the chloroplast genome became a source of DNA markers, which is highly useful for the accurate identification of closely related species [3,7,10], the breeding of fine varieties, and the protection of endangered species [11]. Zhu et al. [7] used “*WCGS*, *CDS*, *mat*K” for cluster analysis of 41 Magnoliaceae plants and found that the results of the three clustering methods were consistent with those of the traditional, morphology-based classification of Magnoliaceae, which provides research background for further accurate identification of Magnoliaceae species and conservation of germplasm resources. By performing comparative study of the chloroplast genomes of *Michelia shiluensis* with four other Magnoliaceae species (*Michelia odora* L., *Magnolia laevifolia* L., *Magnolia insignis* L., and *Magnolia cathcartii* L.) [3], Deng et al. deciphered the relationship between these closely related Magnoliaceae species, determining their classification status. By studying the chloroplast genome of *Magnolia sinostellata*, an endangered Magnoliaceae species, and analyzing the chloroplast SSRs of the closely related species, Yao [11] developed 34 pairs of simple sequence repeat (*SSR*) primers, providing theoretical study for the protection of *M. sinostellata.*

This study involved four species of Magnoliaceae, namely, *Woonyoungia septentrionalis* (Dandy) YW Law, *Michelia champaca* L., *Michelia figo* (Lour.) Spreng, and *Michelia macclurei* Dandy, which are species of broad-leaved Magnoliaceae. Of these four Magnoliaceae species, *M. figo* is an evergreen shrub and the other three are trees. *W. septentrionalis* is an endangered species with dioecious flowers. *M. champaca* is a common garden tree species in southern China. *M. macclurei* is an important timber and tree species for garden and fire-resistant tree species that can be used as a rootstock for related species. Some theoretical bases can be provided for the conservation of endangered magnolia plants through the study of the taxonomic status of *W. septentrionalis* in magnolia plants from the molecular perspective and the phylogenetic relationship between the three common species of magnolia in Guangxi and the endangered species *W. septentrionalis.* This study sequenced the chloroplast genomes of four Magnoliaceae species, obtaining circular sequence maps. Motivated by this, we compared and analyzed their chloroplast genome structure, regional sequence features, and gene distribution of these four species. A CDS-based phylogenetic tree was also constructed. By comparing the chloroplast genomes of twenty related species in GenBank, a phylogenetic analysis of these four magnoliaceae was performed to determine their phylogenetic relationships and analyze their taxonomic status within the magnoliaceae. These results further enrich the chloroplast genome studies of Magnoliaceae species, clarify the taxonomic status of four Magnoliaceae species, and theoretically support future conservation efforts for rare Magnoliaceae plants.

## 2. Materials and Methods

### 2.1. DNA Extraction, Library Construction, Sequencing, and Assembly

Fresh leaves of the Magnoliaceae species (*W. septentrionalis*, *M. champaca*, *M. figo* (Lour.) Spreng, and *M. macclurei*) were collected from a nursery of the College of Forestry, Guangxi University, Nanning, China, and the samples were stored at −80 °C after collection.

Chloroplast genomes of the four Magnoliaceae species were extracted using kits (Plant chloroplast DNA extraction kit RTU5003, Shanghai, China). DNA purity and integrity of four species were determined by 1.0% agarose gel electrophoresis and Nanodrop technique. In order to obtain the original DNA sequence data of the four materials, we commissioned Shenzhen Huitong Biotechnology Co., Ltd., Shenzhen, China for the subsequent library construction, sequencing, assembly, and annotation. The Illumina HiSeq sequencing platform was used for the sequencing, whose quality of the sequencing passed the inspection. We sequenced the DNA profiles of the four species three times to confirm the accuracy of the data. We used de novo assembly to assemble and splice the sequencing results, and SPAdes (3.9.0) [12] (accessed on 1 March 2022; http://cab.spbu.ru/software/spades/) was used for complete assembly of the circular chloroplast genome.

### 2.2. Genome Annotation

We annotated the genetic information of the four chloroplast genomes by comparing them with the CDS of related species. Annotation of chloroplast tRNA was performed online by tRNAscan-SE [13] (accessed on 10 March 2022; http://lowelab.ucsc.edu/tRNAscanSE/platform), the annotation of rRNA was performed online by RNAmmer 1.2 Server [14] (accessed on 11 March 2022; https://services.healthtech.dtu.dk/service.php?RNAmmer-1.2), and the annotation of tmRNA and rnpB was performed by ARAGORN [15] (accessed on 1 March 2022; http://www.ansikte.se/ARAGORN/Downloads/) and Bcheck [16] (accessed on 12 March 2022; http://rna.tbi.univie.ac.at/) online, respectively.

### 2.3. Genome Comparative Analysis

Chloroplast genome maps of the four species were mapped with Organellar genome DRAW (1.3.1) [17] (accessed on 2 April 2022; https://chlorobox.mpimp-golm.mpg.de/OGDraw.html), and DOGMA was used to annotate and draw chloroplast genome linetypes using closely related species as references. Three methods were used: direct blastn comparison, annotation tool DOGMA [17], and selection of chloroplast and bacterial codon tables for ORF prediction and annotation comparison with nr database. mVISTA [18] (accessed on 3 April 2022; https://genome.lbl.gov/vista/index.shtml) was used to compare the sequence difference of four kinds of magnoliaceae plants and *M. champaca* was used as a reference for the sequence alignment. IRscope [19] (accessed on 5 April 2022; https://irscope.shinyapps.io/irapp/) was used for infrared boundary analysis and Mauve (2.4.0) was used for collinearity analysis. Circos (0.69) [20] (accessed on 5 November 2023; http://circos.ca/software/download/circos/) was used to draw circles showing relationships of homology.

### 2.4. SSR Analysis

The Tandem Repeat Finder [21] (accessed on 6 April 2022; https://tandem.bu.edu/trf/trfdesc.html) was used to upload four chloroplast genome sequence files, respectively, and the primary tool was used to carry on the analysis and export the results. Then, we listed each simple repeating sequence in terms of its score, sequence start and stop points, number of segment-specific bases, and sequence of segments.

### 2.5. Codon Bias Analysis

CodonW v 1.4.2 [22] (accessed on 1 May 2022; https://sourceforge.net/projects/codonw/) software was used to analyze the codon usage preference parameters of CDS of the four selected species. The main indexes include amino acid number (L_aa), relative synonymous codon usage (*RSCU*), effective codon number (*ENC*), codon adaptation index (*CAI*), codon preference parameter (*CBI*), *GC*, *GC1*, *GC2*, *GC3*, *GC3s*, and so on. The numbers of *GC1*, *GC2*, and *GC3* represent the number of bits of the codon (1st, 2nd, 3rd), and *GC3s* represent the GC content of the third bit of the synonymous codon. TBtools (0.665) [23] (accessed on 8 May 2022; https://github.com/CJ-Chen/TBtools) was used to plot the hotspot map.

### 2.6. Nucleotide Polymorphism Analysis

For the analysis of magnoliaceae plants, representing four nucleotide polymorphisms in magnoliaceae plants, the DnaSP 6 (12.03) [24] (accessed on 9 May 2022; https://www.softpedia.com/get/Science-CAD/DnaSP.shtml) was used to compare values of Pi(Pi) between the intergenic region and the coding region. For the analysis parameters, the window length was set to 600 and the step size to 200. The difference coefficients were calculated and represented in these plotted images.

### 2.7. Phylogenetic Reconstruction

We performed a phylogenetic analysis using a sequence of twenty-four chloroplast genomes, including four newly sequenced chloroplast genomes from the Magnoliaceae and eighteen others from the Magnoliaceae. Chloroplast genomes of *Liriodendron tulipifera* and *Liriodendron chinensis* were used as controls because both species have been selected for tree construction by scholars conducting phylogenetic study on Magnoliaceae species. Based on relevant research, eighteen other species of magnoliaceae [1,3,8,9,25,26] were selected. Phylotiue (1.2.1) [27] (accessed on 10 May 2022; http://phylosuite.jushengwu.com/) was adopted to perform phylogenetic analysis, while using Bayesian Inference (BI), Maximum Likelihood (MI), and Neighbor-Joining (NJ) to plot the image. The BI tree was reconstructed with GTR+I+G [28]. The ML tree was reconstructed with IQ-TREE [29] and bootstrap probability values were calculated from 1000 replicates. Relevant species annotations were completed using iTOL(6.6) [30] (accessed on 13 May 2022; https://itol.embl.de/). The taxonomic basis of Flora Reipublicae Popularis Sinicae (FRPS) [31,32] was adopted to label the selected magnoliaceae species and compare them with phylogenetic results. Based on a preliminary estimate of the evolutionary steps of the clades [33], we compared the evolutionary relationships of four species of Magnoliaceae. The greater the coefficient of genetic variation, the greater the evolutionary difference and the greater the evolutionary distance. Finally, we used the obtained bootstrap value (*BV*) to evaluate the reliability of each evolutionary branch.

## 3. Results

### 3.1. Chloroplast Genome Sequence and Gene Characteristics Analysis

**Figure 1:**
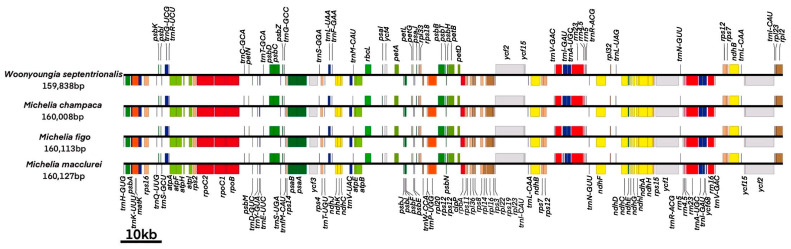
Linear map of chloroplast genomes of the four Magnoliaceae species.

Complete chloroplast genome sequences (NCBI: *Woonyoungia septentrionalis* (Dandy) YW Law: ON456177.1, *Michelia champaca* L.: ON456178.1, *Michelia figo* (Lour.) Spreng: ON456179.1, *Michelia macclurei* Dandy: ON456180.1) of four Magnoliaceae species were obtained. The chloroplast genome sizes of the four Magnoliaceae species were 159,838 bp (*W. septentrionalis*), 160,008 bp (*M. champaca*), 160,113 bp (*M. figo*), and 160,127 bp (*M. macclurei*), respectively (Figure 1 and Table 1). The cpDNAs of the four species typically have four parts, including regions of two *IRs* (26,529–26,602 bp), *LSC* (88,037–88,174 bp), and *SSC* (18,732–18,809 bp). GC contents of four chloroplasts are 39.2% (*M. champaca*, *M. macclurei*) and 39.3% (*W. septentrionalis*, *M. figo*). There are 134 genes in the chloroplast genome of *M. macclurei*, including 89 encoded proteins, 8 rRNAs, and 37 tRNAs. There are 131 genes in the other three chloroplast genomes, including 86 encoded proteins, 8 rRNAs, and 37 tRNAs.

Through genetic analysis, it was found that in four Magnoliaceae plants (Table 2), *atpF*, *ndhA*, *ndhB*, *rpl2, rpoC1, tRNA-Lys*, *tRNA-Ala*, *tRNA-Leu, tRNA-Val, tRNA-Ile,* and *tRNA*-*Gly* genes each had one intron. *ycf3* and *clpP* each contain two introns, and *rsp12* has been identified as a transsplicing gene. The genes *rn4*.5, *rn5*, *rn16*, *rn23*, *tRNA*-*Ile*, *tRNA*-*Arg*, *tRNA*-*Asn*, *tRNA*-*Ala,* and *ORF302* all are two copies in the *IR* region. Among these functional genes, there are six genes involved in ATP synthesis, namely: *atpA*, *atpB*, *atpE*, *atpF*, *atpH,* and *atpI*. There are six genes associated with cytochrome subunits: *petA*, *petB*, *petD*, *petG*, *petL*, and *petN*. There are eleven genes involved in the NADH-dehydrogenase group, five genes involved in the subunit coding of photosystem I, and fifteen genes involved in the subunit synthesis of photosystem II. There are four protein genes with unknown functions, namely: *ycf1*, *ycf2*, *ycf3*, and *ycf4*. There are twelve genes associated with the small ribosome subunits. There are nine genes associated with the large ribosome subunit, and four rRNA genes: *rrn4*.*5*, *rrn5*, *rrn16*, and *rrn23*. There are four genes involved in DNA-dependent RNA polymerase and thirty-five tRNA genes. There are seven other functional genes here: *accD*, *ccsA*, *cemA*, *clpP*, *matK*, *rbcL*, and *infA*. There is also another gene called *ORF302*.

The results showed that after classifying the known functional genes, it was found that there are introns in 19 genes (*W. septentrionalis*), 21 genes (*M. champaca*), 19 genes (*M. figo*), and 20 genes (*M. maclurei*). In terms of intron distribution, there are ten genes in the *LSC* region, four genes in the *IR* region, and one gene in the *SSC* region for the chloroplast genomes of *W. septentrionalis*, *M. figo*, and *M. macclurei*. There are eleven genes in the *LSC* region, four genes in the *IR* region, and two genes in the *SSC* region for the chloroplast genome of *M. macclurei*. In terms of intron length in the chloroplast genomes of the four Magnoliaceae species (Table S3), Group *I* intron of *trnK*-*UUU* is the longest, followed by the intron of *ndhA*. Among the four species, only *ycf3* and *clpP* have Group *II* intron and exon *III*.

### 3.2. Comparative Analysis of Chloroplast Genomes in the Four Magnoliaceae Species

**Figure 2:**
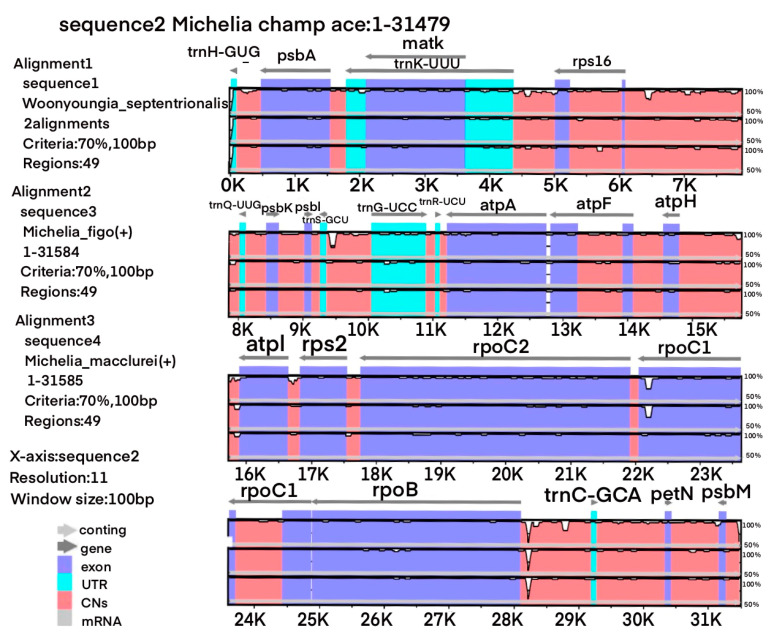
Comparison of the chloroplast genomes of the four Magnoliaceae species using mVISTA. Using *M. champace* as reference, the order of other strips from top to bottom is as follows: *W. septentrionalis*, *M. figo*, *M. macclure*. Above the alignment, gray arrows and thick black lines indicate gene orientations. Purple bars stand for exons, blue bars stand for untranslated regions (UTRs), pink bars stand for noncoding sequences (CNSs), gray bars stand for mRNA, and white peaks stand for differences of genomics. A 70% cutoff identity value was used for the plots. The y-axis represents the percentage identity between 50–100%. The horizontal axis shows the coordinates within the chloroplast genome.

**Figure 3:**
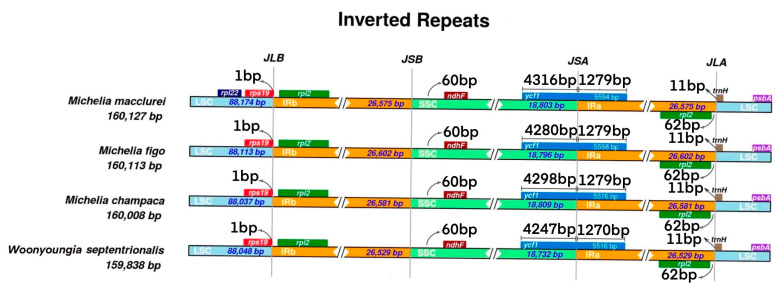
Comparison of large single-copy regions (*LSC*), small single-copy regions (*SSC*), and two inverted repeat regions (*IR*) in the chloroplast genomes of four Magnoliaceae species. The boxes above or below the mainline indicate adjacent boundary genes. This figure is not to scale and only relative changes at or near the IR/SC boundary are shown.

Comparison of chloroplast genomes of the four Magnoliaceae species revealed that they are highly similar (Figure 2 and Figure 3). The length of each region is not significantly different (Table 1). The length of the *LSC* region is 88,037 bp (*M. champaca*) to 88,174 bp (*M. macclurei*), the length of the *SSC* region is 18,732 bp (*W. septentrionalis*) to 18,809 bp (*M. champaca*), and the length of the *IR* region is 26,529 bp (*W. septentrionalis*) to 26,602 bp (*M. figo*).

Regarding the differences among the four regions, the *LSC* region is relatively longer than the *SSC* region and the uncoded region is longer than the coded one. Combined with the results of the *IR* boundary analysis, the highest variation within the coding region of the gene is in *rpoC1*. For regions with different genes, in contrast to the other three species, *M. macclurei* has *rpl22* in the *LSC* region, while *ndhF* is located in the *SSC* region for all four Magnoliaceae species. The *SSC*/*IRa* junction contains the *YCF1* gene in the chloroplasts of four Magnoliaceae species. Both the *rpl2* and *trnH* genes are located at the *IRa*/*LSC* junction, with the *trnH* gene located to the right of the *IRa*/*LSC* junction at a distance of 11 bp and the *rpl2* located to the left of the *IRa*/*LSC* junction. Moreover, *psbA* is located in the *LSC* region. The *rps19* gene is located at the boundary of the *LSC*/*IRb* region, 1bp away from the junction.

**Figure 4:**
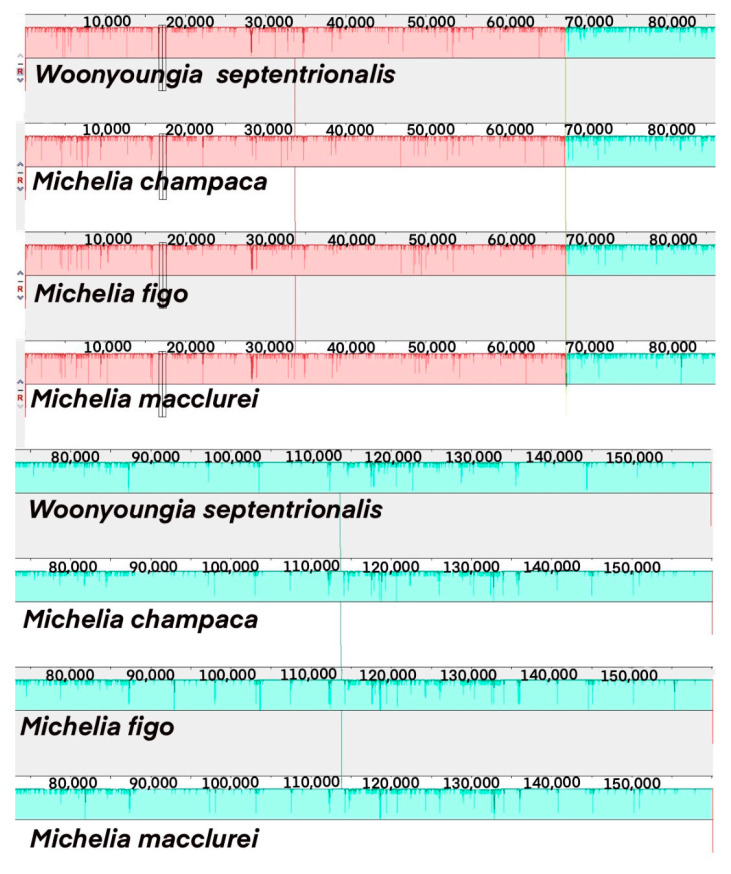
Collinearity analysis of chloroplast genomes of four Magnoliaceae species.

A syntenic analysis of the chloroplast genomes of four Magnoliaceae species revealed high similarity among their chloroplast genome sequences and good synteny (Figure 4). Specifically, the chloroplast genome sequences of *M. figo* were most similar to those of *M. macclurei*, while *M. champaca* showed the lowest similarity with the other three plants’ chloroplast genome sequences.

**Figure 5:**
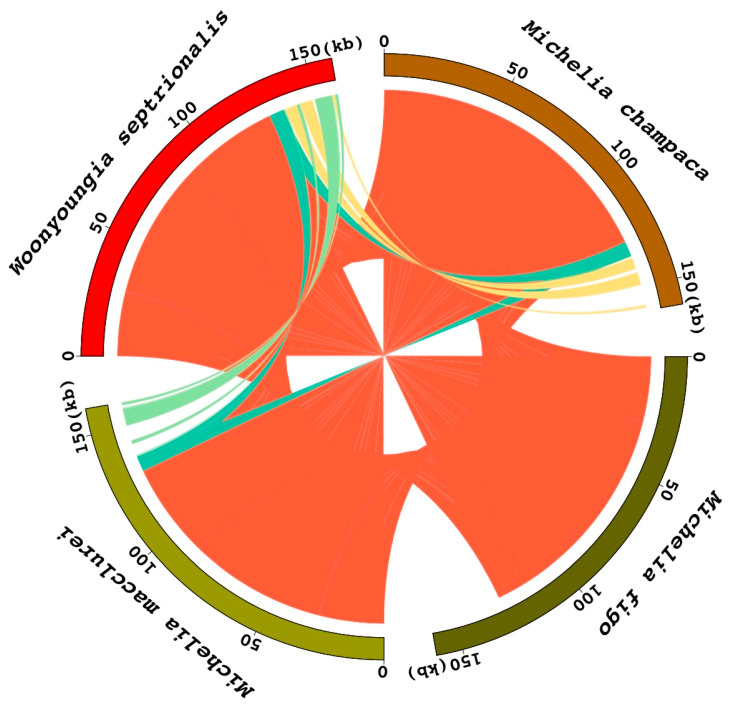
Homology analysis of chloroplast genome sequences in four Magnoliaceae species by Circos. The four outer rings in the graph represent the four sequences and their length (kb). Internal lines represent collinearity between sequences (red represents collinearity between all four sequences, yellow represents collinearity only in *Michelia champaca* and *Woonyoungia septentrionalis*, green represents collinearity only in *Michelia macclurei* and *Woonyoungia septentrionalis*, blue represents collinearity only in *Michelia champaca* and *Michelia macclurei*).

The chloroplast genome sequences of four Magnoliaceae plants were analyzed by homologous comparison, which further confirmed that their chloroplast genome sequences had high similarity (Figure 5). Among them, the chloroplast genome sequences of *M. figo* and the other three species were significantly different. The chloroplast genome sequences of *M. macclurei* and *M. champaca* were relatively different from those of *W. septrionalis*, while the sequences of *M. macclurei* and *M. champaca* were most similar.

### 3.3. SSR Analysis

Four Magnoliaceae species had 95 repeat sequences in total (Supplement Table S2), with one double base *SSR* in *M. figo* (nine in number), and the others compound *SSRS*. *W. septentrionalis* contained 18 repeat sequences, with repeat numbers of 15, 16, 17, 18, 20, 21, 24. *M. champaca* contained 24 repeat sequences, with repeat numbers of repeat sequences of 12, 15, 16, 18, 20, 21, 24, 27. *M. figo* contained 26 repeat sequences, with repeat numbers of repeat sequences of 5, 12, 15, 18, 20, 21, 22, 24, 27. *M. macclurei* contained 27 repeat sequences, with repeat numbers of repeat sequences of 12, 15, 16, 17, 18, 19, 20, 21, 22, 24, 27. Most of these repeats were composed of ATG or ATC bases.

### 3.4. Codon Bias Analysis

**Figure 6:**
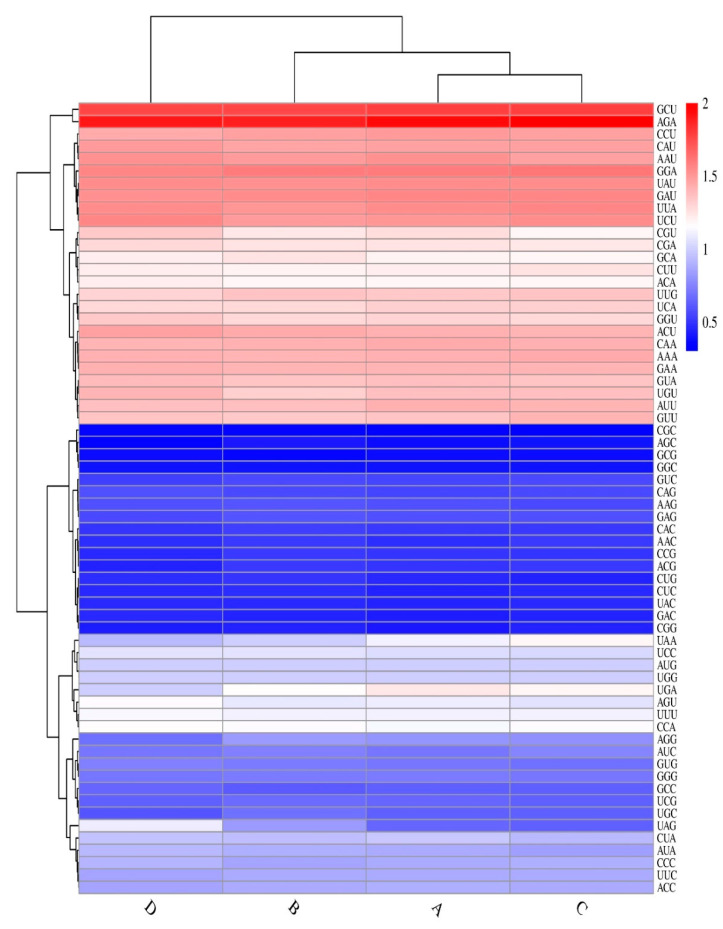
Heat map of usage analysis for four Magnoliaceae chloroplast genomes (A: *W. septentrionalis*, B: *M. champaca*, C: *M. figo*, D: *M. macclure*).

**Figure 7:**
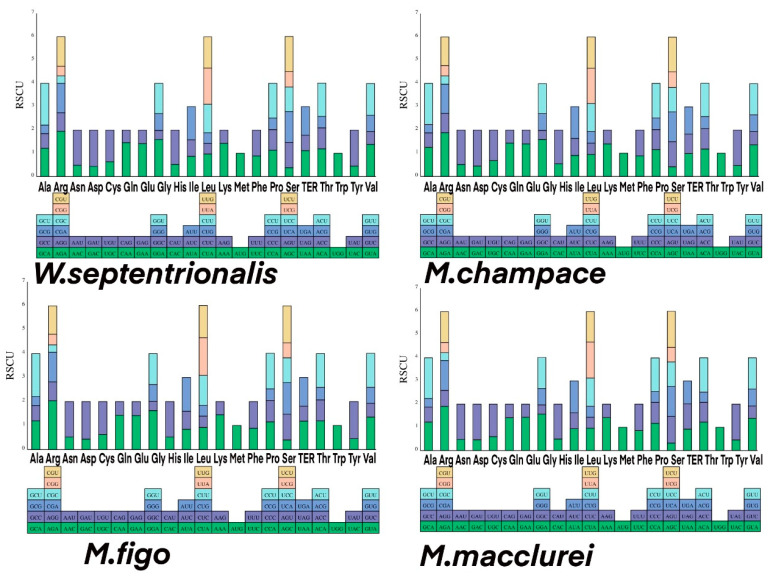
Codon usage in the protein-coding genes of the chloroplast genomes of the four Magnoliaceae species.

The codon composition and *RSCU* in chloroplast genomes of the four Magnoliaceae species (Figure 6 and Figure 7, and Table S1) showed that 33 high-frequency codons have *RSCUs* greater than 1 [34]. Among the high-frequency codons, many have A or U as the third base (15 A, 16 U), but fewer have C or G as the third base (two C, four G). Additionally, the *RSCUs* of the four NCG codons (where N represents any one of the four bases) in chloroplast genomes of the four Magnoliaceae species are relatively low, with GCG in *M. champaca* having the lowest *RSCU* (0.36). The two *NUA*-type codons have a higher *RSCU*, with *UUA* in *M. figo* having the highest value (1.57).

### 3.5. Nucleotide Polymorphism Analysis

**Figure 8:**
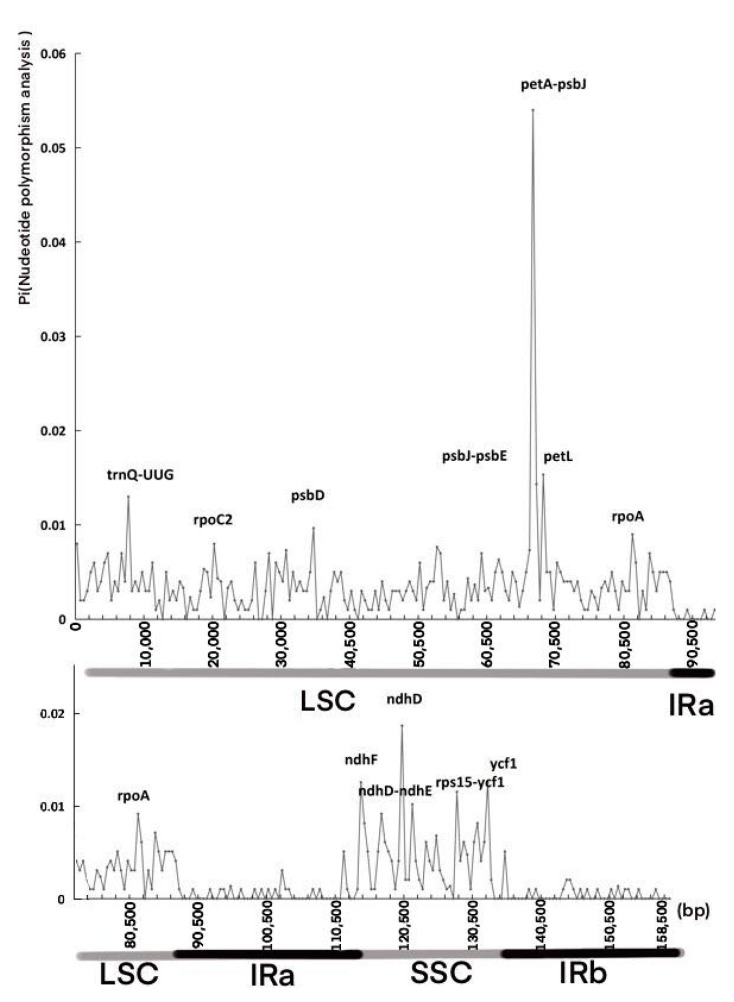
Nucleotide diversity (Pi) values resulting from sliding window analysis of the four Magnoliaceae chloroplast genomes. (*LSC*: large single copy region; IR: inverted repeat regions; *SSC*: small single copy region).

In chloroplast genome studies, mutational hotspots are often used as an important basis for species identification and can provide information about phylogeny [35,36]. Results of nucleotide diversity analysis show that mean Pi in the IR region is lower than that in the *LSC* and *SSC* regions and that the highest nucleotide diversity can be found in the *LSC* region (Figure 8). In the *IR* and *LSC* regions, there are four hotspots, including *petA*-*psbJ* (0.05782), *psbJ*-*psbE* (0.01527), *ndhD*-*ndhE* (0.01091), and *rps15*-*ycf1* (0.012), with a significantly high Pi value (Pi > 0.01), Several other hotspots with a Pi value > 0.01 include *trnQ*-*UUG* (0.01309), *petL* (0.01636), *ndhF* (0.01309), *ndhD* (0.01745), and *ycf1* (0.01309). However, in the *SSR* region, none of the hotspots has a significantly high Pi value (Pi > 0.01). The mean nucleotide diversity (Pi) of the four Magnoliaceae species is 0.00283, and the hotspot petA-psbJ (0.05782) has the highest Pi value (>0.05).

### 3.6. Phylogenetic Analysis of the Four Magnoliaceae Species

**Figure 9:**
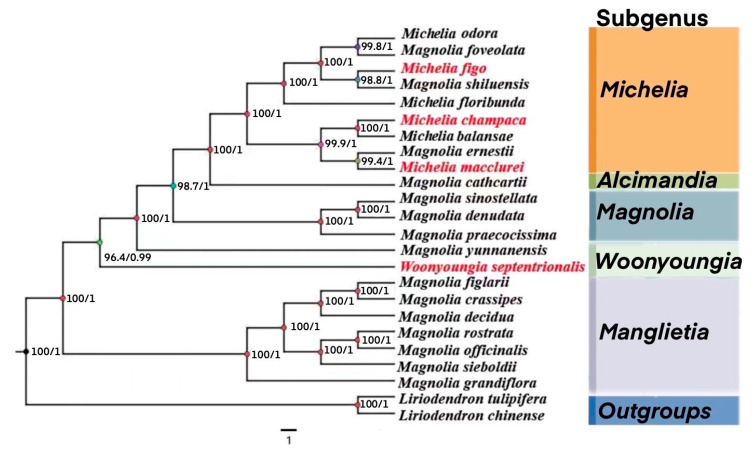
Phylogenetic analysis of 24 magnolia species based on the Neighbor-Joining (NJ) method. *Liriodendron tulipifera* and *Liriodendron chinense* are used as outgroups. Four Magnoliaceae species were shown in red. The node in Figure 9 is the value of BVs.

The phylogenetic evolution of twenty-four magnolia chloroplast genomes (species names and GenBank entry numbers are shown in Table 3, phylogenetic locations are shown in Figure 9, Figures S1 and S2) was analyzed, including four magnolia species with chloroplast gene sequences measured in this study, eighteen additional magnolia species, and two exomorphs. Based on the CDS clustering results, these chloroplast genome exclusions contain five subgenera (*Michelia, Alcimandia*, *Magnolia*, *Woonyoungia,* and *Manglietia*), and the three methods yielded consistent results. *W. septentrionalis* is further away from the other three species, all of which cluster towards Michaelia. Where *W. septentrionalis* and *M. yunnanensis* are most closely related, they are grouped together as *Woonyoungia*, *M. figo,* and *Magnolia shiluensis*, so it is the same with *M. champaca* and *Michelia balansae* as well as *M. macclurei* and *Magnolia ernestii* which come together. Except for the individual cases mentioned above, the clustering results of other magnolia species were consistent with those of the Plant wisdom (http://www.iplant.cn/) and the NCBI database classification system. Liriodendron contains *Liriodendron tulipifera* and *Liriodendron chinense,* which cluster together, and the bootstrap value at the nodule is 100, which is consistent with the research results of Salvador Guzman-Diaz et al. [1]. In the full phylogenetic relation results of most nodules, the bootstrap value is above 95, indicating high reliability of clustering results.

## 4. Discussion

The four Magnoliaceae species whose chloroplast genomes are approximately 159–160 kb in size are genetically and structurally similar, and their observed gene sequences in all genomes have high synteny. Furthermore, the four species have a similar GC content, which directly affects codon usage [37]. Chloroplast genome structure and GC content suggest that these four Magnoliaceae species may share a close phylogenetic relationship.

Chloroplast genomes of Magnoliaceae plants are highly conserved, and this study identified gene loss, duplication, and intron loss. The commonly reported gene losses in angiosperm cpDNAs (*rpl2*, *accD*, *ndhF*, *psbE*, *rpl23*, *trnL*-*CCA*, *trnG*-*GCC*) [38,39,40] were identified in the cpDNAs of the four Magnoliaceae species. For the four Magnoliaceae species, the *IR*/*SC* boundary region exhibits similar characteristics, slight differences are observed in the lengths of the genes on both sides of the junction and the distances between different genes at the junction, and the expansion of the *IR* region results in changes in chloroplast genome length. However, the size of the whole chloroplast genome does not always increase with the expansion of *IR* [41]. For example, among the four Magnoliaceae species, *M. figo* has the largest *IR* (26,602 bp), but the whole genome size (160,113 bp) is second to that of *M. macclurei* (160,127 bp); this result has also been obtained in similar studies by other scholars [3,42,43] due to the specific differences that exist between species. Therefore, the contraction and expansion of the *IR* region of the cp genome are considered important evolutionary phenomenon [44], which may lead to size changes in the chloroplast genome, the generation of pseudogenes, gene duplication, or the reduction of replicative genes to a single copy [45,46].

In addition, through homologous comparison of chloroplast genome sequences, we have preliminarily speculated that *M. figo* and *W. septentrionalis* are most closely related, and *M. champace* and *M. macclurei* are most closely related, based on sequence similarity.

We detected a total of 95 *SSR* sequences in the chloroplast genomes of the four magnoliaceae. In *M. figo* there were only a few simple repeats, while the other three species were basically similar, all of which were predominantly composed of complex repeats. The higher ratios of A/T and TA/TA suggest higher genetic diversity in these regions [47,48]. No rearrangements were found in the four species, possibly due to the lack of large-scale complex repeats (>100 bp), which is consistent with previous studies on other species (*Magnolia grandiflora*, *Magnolia zenii*) [49,50]. The analysis of *SSR* in the chloroplast genomes of these four plants provides a potential application prospect for molecular marker research on Magnoliaceae in the future.

Based on chloroplast genomes from the four Magnoliaceae species, mutational hotspots were identified within *CDS* and noncoding regions. The nucleotide diversity (Pi) of the hotspot region *petA*-*psbJ* reaches 0.05782, significantly higher than that of the common barcode gene *rbcL* (0.02149) [50,51,52], indicating the potential of *petA*-*psbJ* as a barcode region for identifying Magnoliaceae species. In the *SSC* region, *ndhD* is the most variable gene, showing better discriminative ability than *petL* and *ycf1*, and these highly variable regions can be applied to the identification or phylogenetic analysis of Magnoliaceae species [53].

The phylogenetic relationships and taxonomic status of some magnoliaceae species remain unclear [54,55]. There is limited molecular data available about them as well. For example, in this study, *W. septentrionalis* was magnolia, and *Magnolia yunnanensis* was magnolia, but they were clustered together (bootstrap = 100). Before 2012, scholars classified *M. yunnanensis* into Woonyoungia [55], but based on the molecular findings, the taxonomic relationship between the two might now be questionable. The taxonomic status of *M. yunnanensis* is also open to debate. Additionally, questions arise regarding the phylogenetic relationship between *Magnolia Shiluenis*–*M. figo* clade (bootstrap = 98.8) and *Magnolia ernestii–Michelia macclurei* clade (bootstrap = 99.4), which do not belong to the same genus in traditional taxonomic status. This result could be caused by the differences between nuclear inheritance and plasmid inheritance, warranting further discussion [5]. We have been able to determine the relationship between four species whereby *M*. *figo* is the most distant from *W. septentrionalis* and *M. champaca* is the closest from *M. macclurei*. Additionally, the phylogenetic relationships of *Magnolia sinostellata*–*Magnolia praecocissima* and *Magnolia figlarii*–*Magnolia granfiflora* (bootstrap = 100) were also identified in the analysis. In this study, only four genera, Woonyoungia, Michelia, Magnolia, and Liriodendron, were selected for phylogenetic analysis based on the *CDS*, and the results showed that the first three genera were far more closely related to Liriodendron. The phylogenetic relationships of these 24 species of Magnoliaceae were identified, and their clustering was consistent with FRPS data (http://www.iplant.cn/). The phylogenetic location of *W. septentrionalis* found in this study is consistent with the findings of other scholars [50,56], and the evolutionary locations of *W. septentrionalis* and *M. champaca* are consistent with those reported by S. Chen et al. [57]. The relatively short genetic distance between *M. macclurei*, *M. champaca,* and *M. figo* suggests close phylogenetic relationships among them, whereby *M. figo* could serve as a breeding population for endangered and less resistant magnoliaceae species. The BV of all four species of Magnoliaceae was above 90, providing high reliability for the results. *M. yunnanensis* is close to *W*. *septentrionalis*, both of which are endangered species of Magnoliaceae. Liriodendron includes *L*. *tulipifera* and *L*. *chinense*, which are clustered together and have distant evolutionary relations with other magnoliaceae species, consistent with the findings of Salvador Guzmann-Diaz et al. [1,25,58,59].

## 5. Conclusions

In this study, chloroplast genomes of four Magnoliaceae species were reported and subjected to a *CDS*-based phylogenetic comparative analysis with other published Magnoliaceae species. Chloroplast genome structure and gene content of the four Magnoliaceae species are similar, with high conservation and a high degree of methylation. The hotspot region (*petA*-*psbJ*) can be used as a potential molecular marker for the identification of Magnoliaceae plants. The degree of evolution for the four Magnoliaceae species from low to high is *W. septentrionalis*, *M. champaca, M. macclurei,* and *M. figo*. Phylogenetic tree analysis showed that 24 Magnoliaceae species completely clustered into 11 genera. This study provides additional data to help to solve evolutionary complexities within the family Magnoliaceae. It also can enable the development of genetic markers for species identification in the future.

## Figures and Tables

**Table 1:** Basic information of chloroplast genomes of the four Magnoliaceae species.

	*M. macclurei*	*W. septentrionalis*	*M. champaca*	*M. figo*
Total length (bp)	160,127	159,838	160,008	160,113
LSC length (bp)	88,174	88,048	88,037	88,113
SSC length (bp)	18,803	18,732	18,809	18,796
IR length (bp)	26,575	26,529	26,581	26,602
GC content (%)	39.2%	39.3%	39.2%	39.3%
Total	134	131	131	131
Protein coding genes	89	86	86	86
rRNA	8	8	8	8
tRNA	37	37	37	37

**Table 2:** Genes contained in the chloroplast genomes of four Magnoliaceae species. (* genes with one intron, ** genes with two introns, # transsplice genes, and one gene with two copies in the IR region).

Gene Function	Gene Names
ATP synthase	*atpA*, *atpB*, *atpE*, atpF *, *atpH*, *atpI*
Cytochrome b/f complex	*petA*, *petB*, *petD*, *petG*, *petL*, *petN*
NADH dehydrogenase	ndhA *, ndhB *, *ndhC*, *ndhD*, *ndhE*, *ndhF*,*ndhG*, *ndhH*, *ndhI*, *ndhJ*, *ndhK*
Photosystem I	*psaA*, *psaB*, *psaC*, *psaI*, *psaJ*
Photosystem II	*psbA*, *psbB*, *psbC*, *psbD*, *psbE*, *psbF*,*psbH*, *psbI*, *psbJ*, *psbK*, *psbL*, *psbM*, *psbN*, *psbT*, *psbZ*
Proteins of unknown function	*ycf11*, *ycf2*, ycf3 **, *ycf4*
Ribosomal proteins (SSU)	*rps2*, *rps3*, *rps4*, *rps7*, *rps8*, *rps11*, *rps12 #*, *rps14*, *rps15*, *rps16*, *rps18*, *rps19*
Ribosomal proteins (LSU)	rpl2 *, *rpl14*, *rpl16*, *rpl20*, *rpl22*, *rpl23*, *rpl32*, *rpl33*, *rpl36*
Ribosomal RNAs	*rrn4.51*, *rrn51*, *rrn161*, *rrn231*
RNA polymerase	*rpoA*, *rpoB*, rpoC1 *, *rpoC2*
Other genes	*accD*, *ccsA*, *cemA*, clpP **, *matK*, *rbcL*, *infA*
Transfer RNAs	tRNA-Lys *, *tRNA-Gln*, *tRNA-Ser*, tRNA-Gly *, *tRNA-Arg*, *tRNA-Cys*, *tRNA-Asp*, *tRNA-Tyr*, *tRNA-Glu*, *tRNA-Thr*, *tRNA-Ser*, *tRNA-Gly*, *tRNA-Met*, *tRNA-Ser*, *tRNA-Thr*, *tRNA-Leu*, *tRNA-Phe*, *tRNA-Val*, *tRNA-Met*, *tRNA-Trp*, *tRNA-Pro*, *tRNA-Ile*, tRNA-Leu*, tRNA-Val *, *tRNA-His*, tRNA-Ile *1, tRNA-Ala *1, *tRNA-Arg1*, *tRNA-Asn1*, *tRNA-Leu*, *tRNA-Asn*, *tRNA-Arg*, *tRNA-Ala*, *tRNA-Ile*, *tRNA-His*
Other	*ORF3021*

**Table 3:** Chloroplast genome features of selected Magnoliaceae species from NCBI.

Species	Genome Size/bp	GC Content/%	Accession Number
*M* *ichelia figo*	160,113	39.3	ON 456179.1
*Magnolia shiluensis*	160,075	39.3	NC_047417.1
*Michelia odora*	160,070	39.3	NC_023239.1
*Magnolia foveolata*	160,082	39.3	NC_062644.1
*Michelia flloribunda*	160,049	39.2	MN 897728.1
*Michelia maccurei*	160,127	39.2	ON 456180.1
*Magnolia ernestii*	160,100	39.2	MN 897729.1
*Michelia champaca*	160,008	39.2	ON 456178.1
*Magnolia balansae*	160,134	39.2	NC_053860.1
*Magnolia cathcartii*	159,926	39.2	MZ 329179.1
*Magnolia sinostellata*	160,076	39.2	NC_039941.1
*Magnolia denudata*	160,090	39.2	NC_056770.1
*Magnolia praecocissima*	159,778	39.3	NC 058268.1
*Magnolia yunnanensis*	160,085	39.3	KF 753638.1
*Woonyoungia septentrionalis*	159,838	39.3	ON 456177.1
*Magnolia rostrata*	160,070	39.3	NC_058997.1
*Magnolia officinalis*	160,009	39.2	MW373503.1
*Magnolia sieboldii*	160,177	39.2	NC_041435.1
*Magnolia figlarii*	160,120	39.3	MT 682874.1
*Magnolia crassipes*	159,901	39.3	NC_058270.1
*Magnolia decidua*	160,127	39.3	NC_062919.1
*Magnolia grandiflora*	159,623	39.3	NC_020318.1
*Liriodendron chinense*	159,611	39.2	MK 887907.1
*Liriodendron tulipifera*	159,886	39.2	NC_008326.1

## Data Availability

The datasets generated during the current study are available in the [NCBI] (accessed on 10 May 2022) repository, the assembled chloroplast genomes of four Magnoliaceae species were deposited in GenBank form: *Woonyoungia septentrionalis* (Dandy) YW Law: ON456177.1 (https://www.ncbi.nlm.nih.gov/nuccore/ON456177.1/), *Michelia champaca* L.: ON456178.1 (https://www.ncbi.nlm.nih.gov/nuccore/ON456178.1/), *Michelia figo* (Lour.) Spreng: ON456179.1 (https://www.ncbi.nlm.nih.gov/nuccore/ON456179.1/), *Michelia macclurei* Dandy: ON456180.1 (https://www.ncbi.nlm.nih.gov/nuccore/ON456180.1/).

## Supplementary Materials

The following supporting information can be downloaded at: https://www.mdpi.com/article/10.3390/cimb45110578/s1, Figure S1: Phylogenetic analysis of 24 Magnoliaceae species based on Maximum Likelihood(ML) inference; Figure S2: Phylogenetic analysis of 24 magnolia species obtained from new Bayesian Inference(BI) analysis; Table S1: Statistical analysis of chloroplast genome codon of four Magnolia species; Table S2: Analysis of simple sequence repeats (SSRs) in four Magnolia chloroplast genomes; Length of exons and introns in genes with introns in the Magnolia chloroplast genome. Table S3: Length of exons and introns in genes with introns in the Magnolia chloroplast genome.

## References

[1] Guzmán-Díaz S., Núñez F.A.A., Veltjen E., Asselman P., Larridon I., Samain M.S. (2022). Comparison of *Magnoliaceae plastomes*: Adding Neotropical *Magnolia* to the Discussion. Plants.

[2] Daniell H., Lin C.S., Yu M., Chang W.J. (2016). Chloroplast genomes: Diversity, evolution, and applications in genetic engineering. Genome Biol..

[3] Deng Y., Luo Y., He Y., Qin X., Li C., Deng X. (2020). Complete chloroplast genome of *Michelia shiluensis* and a comparative analysis with four *Magnoliaceae* species. Forests.

[4] Park J., Xi H., Kim Y. (2020). The Complete Chloroplast Genome of *Arabidopsis thaliana* Isolated in Korea (Brassicaceae): An Investigation of Intraspecific Variations of the Chloroplast Genome of Korean *A. thaliana*. Int. J. Genom..

[5] Zhang Y., Tian L., Lu C. (2023). Chloroplast gene expression: Recent advances and perspectives. Plant Commun..

[6] Perumal S., Waminal N.E., Lee J., Koo H.J., Choi B.S., Park J.Y., Yang T.J. (2021). Nuclear and chloroplast genome diversity revealed by low-coverage whole-genome shotgun sequence in 44 Brassica oleracea breeding lines. Hortic. Plant J..

[7] Zhu B., Qian F., Wang X.S., Liu Y.L. (2022). The phylogeny of *Magnoliaceae* based on chloroplast genome. J. Biol..

[8] Kaikai J.I., Xiqiang S., Chunguo C. (2020). Codon Usage Profiling of Chloroplast Genome in *Magnoliaceae*. J. Agric. Sci. Technol..

[9] Yang F., Cai L., Dao Z., Sun W. (2022). Genomic Data Reveals Population Genetic and Demographic History of *Magnolia fistulosa* (*Magnoliaceae*), a Plant Species with Extremely Small Populations in Yunnan Province, China. Front. Plant Sci..

[10] Li D.M., Zhu G.F., Xu Y.C., Ye Y.J., Liu J.M. (2020). Complete Chloroplast Genomes of Three *Medicinal Alpinia* Species: Genome Organization, Comparative Analyses and Phylogenetic Relationships in Family Zingiberaceae. Plants.

[11] Yao Z.X. (2019). The Conservation Genetics Research of *Magnolia sinostellata*. Ph.D. Thesis.

[12] Li Z., Chen Y., Mu D., Yuan J., Shi Y., Zhang H., Gan J., Li N., Hu X., Liu B. (2012). Comparison of the two major classes of assembly algorithms: Overlap-layout-consensus and de-bruijn-graph. Brief. Funct. Genom..

[13] Schattner P., Brooks A.N., Lowe T.M. (2005). The tRNAscan-SE, snoscan and snoGPS web servers for the detection of tRNAs and snoRNAs. Nucleic Acids Res..

[14] Lagesen K., Hallin P., Rødland E.A., Stærfeldt H.H., Rognes T., Ussery D.W. (2007). RNAmmer: Consistent and rapid annotation of ribosomal RNA genes. Nucleic Acids Res..

[15] Laslett D., Canback B. (2004). ARAGORN, a program to detect tRNA genes and tmRNA genes in nucleotide sequences. Nucleic Acids Res..

[16] Ellis J.C. (2020). P finder: Genomic and metagenomic annotation of RNase P RNA gene (*rnpB*). BMC Genom..

[17] Lohse M., Drechsel O., Kahlau S., Bock R. (2013). OrganellarGenomeDRAW—A suite of tools for generating physical maps of plastid and mitochondrial genomes and visualizing expression data sets. Nucleic Acids Res..

[18] Zhang X., Gu C., Zhang T., Tong B., Zhang H., Wu Y., Yang C. (2020). Chloroplast (Cp) Transcriptome of *P. davidiana* Dode×P. bolleana Lauch provides insight into the Cp drought response and Populus Cp phylogeny. BMC Evol. Biol..

[19] Zheng S., Poczai P., Hyvönen J., Tang J., Amiryousefi A. (2020). Chloroplot: An Online Program for the Versatile Plotting of Organelle Genomes. Front. Genet..

[20] Krzywinski M., Schein J., Birol I., Connors J., Gascoyne R., Horsman D., Jones S.J., Marra M.A. (2009). Circos: An information aesthetic for comparative genomics. Genome Res..

[21] Shi L., Chen H., Jiang M., Wang L., Wu X., Huang L., Liu C. (2019). CPGAVAS2, an integrated plastome sequence annotator and analyzer. Nucleic Acids Res..

[22] Wang P., Mao Y., Su Y., Wang J. (2021). Comparative analysis of transcriptomic data shows the effects of multiple evolutionary selection processes on codon usage in *Marsupenaeus japonicus* and *Marsupenaeus pulchricaudatus*. BMC Genom..

[23] Chen C., Chen H., Zhang Y., Thomas H.R., Frank M.H., He Y., Xia R. (2020). TBtools: An Integrative Toolkit Developed for Interactive Analyses of Big Biological Data. Mol. Plant.

[24] Rozas J., Ferrer-Mata A., Sánchez-DelBarrio J.C., Guirao-Rico S., Librado P., Ramos-Onsins S.E., Sánchez-Gracia A. (2017). DnaSP 6: DNA Sequence Polymorphism Analysis of Large Data Sets. Mol. Biol. Evol..

[25] Liu G.N., Liu B.B., Wen J., Wang Y.B. (2020). The complete chloroplast genome sequence of *Magnolia mexicana* DC. (*Magnoliaceae*) from Central America. Mitochondrial DNA Part B.

[26] Park J., Kim Y., Kwon W., Xi H., Kwon M. (2019). The complete chloroplast genome of tulip tree, *Liriodendron tulifipera* L. (*Magnoliaceae*): Investigation of intra-species chloroplast variations. Mitochondrial DNA Part B Resour..

[27] Zhang D., Gao F., Jakovlić I., Zou H., Zhang J., Li W.X., Wang G.T. (2020). PhyloSuite: An integrated and scalable desktop platform for streamlined molecular sequence data management and evolutionary phylogenetics studies. Mol. Ecol. Resour..

[28] Abadi S., Azouri D., Pupko T., Mayrose I. (2019). Model selection may not be a mandatory step for phylogeny reconstruction. Nat. Commun..

[29] Minh B.Q., Schmidt H.A., Chernomor O., Schrempf D., Woodhams M.D., von Haeseler A., Lanfear R. (2020). IQ-TREE 2: New Models and Efficient Methods for Phylogenetic Inference in the Genomic Era. Mol. Biol. Evol..

[30] Letunic I., Bork P. (2021). Interactive Tree of Life (iTOL) v5: An online tool for phylogenetic tree display and annotation. Nucleic Acids Res..

[31] Magnoliaceae in Flora of China @Efloras.Org. http://efloras.org/florataxon.aspx?flora_id=2&taxon_id=10530.

[32] Figlar R.B. A Brief Taxonomic History of Magnolia. https://www.magnoliasociety.org/ClassificationArticle.

[33] Ford C.S., Ayres K.L., Toomey N., Haider N., Van Alphen Stahl J., Kelly L.J., Wikström N., Hollingsworth P.M., Duff R.J., Hoot S.B. (2009). Selection of candidate coding DNA barcoding regions for use on land plants. Bot. J. Linn. Soc..

[34] Wu X.M., Wu S.F., Ren D.M., Zhu Y.P., He F.C. (2007). The analysis method and progress in the study of codon bias. Yi ChuanHereditas.

[35] Zhou Y., Nie J., Xiao L., Hu Z., Wang B. (2018). Comparative Chloroplast Genome Analysis of Rhubarb Botanical Origins and the Development of Specific Identification Markers. Molecules.

[36] Niu Z., Xue Q., Zhu S., Sun J., Liu W., Ding X. (2017). The Complete Plastome Sequences of Four *Orchid* Species: Insights into the Evolution of the Orchidaceae and the Utility of Plastomic Mutational Hotspots. Front. Plant Sci..

[37] Knight R.D., Freeland S.J., Landweber L.F. (2001). A simple model based on mutation and selection explains trends in codon and amino-acid usage and GC composition within and across genomes. Genome Biol..

[38] Liu J., Jiang M., Chen H., Liu Y., Liu C., Wu W. (2021). Comparative genome analysis revealed gene inversions, boundary expansions and contractions, and gene loss in the *Stemona sessilifolia* (Miq.) Miq. Chloroplast genome. PLoS ONE.

[39] Xiao-Ming Z., Junrui W., Li F., Sha L., Hongbo P., Lan Q., Jing L., Yan S., Weihua Q., Lifang Z. (2017). Inferring the evolutionary mechanism of the chloroplast genome size by comparing whole-chloroplast genome sequences in seed plants. Sci. Rep..

[40] Chris Blazier J., Guisinger M.M., Jansen R.K. (2011). Recent loss of plastid-encoded *ndh* genes within *Erodium* (Geraniaceae). Plant Mol. Biol..

[41] She H., Liu Z., Xu Z., Zhang H., Cheng F., Wu J., Qian W. (2022). Comparative chloroplast genome analyses of cultivated spinach and two wild progenitors shed light on the phylogenetic relationships and variation. Sci. Rep..

[42] He S., Yang Y., Li Z., Wang X., Guo Y., Wu H. (2020). Comparative analysis of four *Zantedeschia* chloroplast genomes: Expansion and contraction of the IR region, phylogenetic analyses and SSR genetic diversity assessment. PeerJ.

[43] Li D.M., Li J., Wang D.R., Xu Y.C., Zhu G.F. (2021). Molecular evolution of chloroplast genomes in subfamily Zingiberoideae (Zingiberaceae). BMC Plant Biol..

[44] Nazareno A.G., Carlsen M., Lohmann L.G. (2015). Complete Chloroplast Genome of *Tanaecium tetragonolobum*: The First Bignoniaceae Plastome. PLoS ONE.

[45] Liang H., Zhang Y., Deng J., Gao G., Ding C., Zhang L., Yang R. (2020). The complete chloroplast genome sequences of 14 *Curcuma* species: Insights into genome evolution and phylogenetic relationships within Zingiberales. Front. Genet..

[46] Abdullah, Mehmood F., Shahzadi I., Waseem S., Mirza B., Ahmed I., Waheed M.T. (2020). Chloroplast genome of *Hibiscus rosa-sinensis* (Malvaceae): Comparative analyses and identification of mutational hotspots. Genomics.

[47] Chen Z., Hu F., Wang X., Fan H., Zhang Z. (2017). Analysis of codon usage bias of *Ananas comosus* with genome sequencing data. J. Fruit Sci..

[48] Al-Saif M., Khabar K.S. (2012). UU/UA dinucleotide frequency reduction in coding regions results in increased mRNA stability and protein expression. Mol. Ther. J. Am. Soc. Gene Ther..

[49] Li X., Gao H., Wang Y., Song J., Henry R., Wu H., Hu Z., Yao H., Luo H., Luo K. (2013). Complete chloroplast genome sequence of *Magnolia grandiflora* and comparative analysis with related species. Sci. China Life Sci..

[50] Li Y., Sylvester S.P., Li M., Zhang C., Li X., Duan Y., Wang X. (2019). The Complete Plastid Genome of *Magnolia zenii* and Genetic Comparison to *Magnoliaceae* species. Molecules.

[51] Jamdade R., Upadhyay M., Al Shaer K., Al Harthi E., Al Sallani M., Al Jasmi M., Al Ketbi A. (2021). Evaluation of *Arabian Vascular* Plant Barcodes (*rbc*L and *mat*K): Precision of Unsupervised and Supervised Learning Methods towards Accurate Identification. Plants.

[52] Fujii T., Ueno K., Shirako T., Nakamura M., Minami M. (2022). Identification of Lagopus muta japonica food plant resources in the Northern Japan Alps using DNA metabarcoding. PLoS ONE.

[53] Dong S., Zhou M., Zhu J., Wang Q., Ge Y., Cheng R. (2022). The complete chloroplast genomes of *Tetrastigma hemsleyanum* (Vitaceae) from different regions of China: Molecular structure, comparative analysis and development of DNA barcodes for its geographical origin discrimination. BMC Genom..

[54] Callaghan C.B., Png S.K. (2020). Twenty-six additional new combinations in the *Magnolia* (*Magnoliaceae*) of China and Vietnam. PhytoKeys.

[55] Hinsinger D.D., Strijk J.S. (2017). The chloroplast genome sequence of *Michelia alba* (*Magnoliaceae*), an ornamental tree species. Mitochondrial DNA. Part B Resour..

[56] Wang Y.B., Liu B.B., Nie Z.L., Chen H.F., Chen F.J., Figlar R.B., Wen J. (2020). Major clades and a revised classification of *Magnolia* and *Magnoliaceae* based on whole plastid genome sequences via genome skimming. J. Syst. Evol..

[57] Chen S., Wu T., Fu Y., Hao J., Ma H., Zhu Y., Sima Y. (2020). Complete chloroplast genome sequence of *Michelia champaca* var. champaca Linnaeus, an ornamental tree species of *Magnoliaceae*. Mitochondrial DNA Part B Resour..

[58] Wei S., Luo S., Zhong Y., Zhou Y., Song Z. (2022). The complete chloroplast genome sequence of *Michelia macclurei* (Dandy, 1928) (*Magnoliaceae*), an important fire-resistant tree species. Mitochondrial DNA Part B Resour..

[59] Yang L., Tian J., Xu L., Zhao X., Song Y., Wang D. (2022). Comparative Chloroplast Genomes of Six *Magnoliaceae* Species Provide New Insights into Intergeneric Relationships and Phylogeny. Biology.

