# Supplementary material for: Phylogenetics Study to Compare Chloroplast Genomes in Four Magnoliaceae Species
Source: Curr Issues Mol Biol. 2023 Nov 16;45(11):9234–51. doi: 10.3390/cimb45110578 (PMC10670740; doi:10.3390/cimb45110578)
Supplement: Supplementary file 1 [file cimb-45-00578-s001.zip › cimb-2683688-supplementary.pdf]

Supplementary information

Table S1. Statistical analysis of chloroplast genome codon of four Magnolia species.

**RSCU**		***W. septentrionalis***	***M. champaca***	***M. figo***	***M. macclurei***
Phe	UUU	1.12	1.12	1.12	1.14
	UUC	0.88	0.88	0.88	0.86
Leu	UUA	1.55	1.51	1.57	1.55
	UUG	1.34	1.35	1.35	1.31
	CUU	1.22	1.21	1.25	1.22
	CUC	0.45	0.48	0.47	0.47
	CUA	0.97	0.95	0.92	0.96
	CUG	0.46	0.5	0.45	0.48
Ile	AUU	1.42	1.37	1.41	1.37
	AUC	0.71	0.73	0.75	0.7
	AUA	0.87	0.9	0.84	0.93
Met	AUG	1	1	1	1
Val	GUU	1.36	1.34	1.41	1.35
	GUC	0.55	0.57	0.57	0.53
	GUA	1.38	1.36	1.35	1.39
	GUG	0.7	0.72	0.68	0.73
Ser	UCU	1.51	1.49	1.55	1.56
	UCC	1.05	1.06	1.03	1.06
	UCA	1.32	1.28	1.32	1.29
	UCG	0.66	0.67	0.63	0.63
Pro	CCU	1.49	1.48	1.48	1.45
	CCC	0.88	0.86	0.89	0.91
	CCA	1.13	1.15	1.15	1.17
	CCG	0.5	0.51	0.49	0.46
Thr	ACU	1.43	1.44	1.41	1.48
	ACC	0.9	0.88	0.88	0.86
	ACA	1.19	1.18	1.19	1.22
	ACG	0.49	0.51	0.52	0.44
Ala	GCU	1.79	1.77	1.79	1.76
	GCC	0.63	0.61	0.64	0.65
	GCA	1.21	1.26	1.19	1.22
	GCG	0.37	0.36	0.38	0.37
Tyr	UAU	1.55	1.53	1.54	1.54

	UAC	0.45	0.47	0.46	0.46
TER	UAA	1.11	0.99	1.18	0.92
	UAG	0.66	0.83	0.63	1.09
His	CAU	1.48	1.46	1.48	1.5
	CAC	0.52	0.54	0.52	0.5
Gln	CAA	1.45	1.43	1.43	1.41
	CAG	0.55	0.57	0.57	0.59
Asn	AAU	1.52	1.49	1.48	1.52
	AAC	0.48	0.51	0.52	0.48
Lys	AAA	1.42	1.4	1.44	1.42
	AAG	0.58	0.6	0.56	0.58
Asp	GAU	1.57	1.55	1.56	1.53
	GAC	0.43	0.45	0.44	0.47
Glu	GAA	1.41	1.4	1.41	1.43
	GAG	0.59	0.6	0.59	0.57
Cys	UGU	1.37	1.32	1.37	1.4
	UGC	0.63	0.68	0.63	0.6
TER	UGA	1.23	1.17	1.19	0.99
Trp	UGG	1	1	1	1
Arg	CGU	1.27	1.23	1.19	1.34
	CGC	0.32	0.35	0.31	0.34
	CGA	1.26	1.26	1.24	1.29
	CGG	0.41	0.45	0.44	0.43
Ser	AGU	1.09	1.08	1.07	1.15
	AGC	0.38	0.41	0.4	0.32
Arg	AGA	1.94	1.88	2.03	1.9
	AGG	0.8	0.83	0.79	0.69
Gly	GGU	1.3	1.29	1.29	1.34
	GGC	0.39	0.39	0.39	0.39
	GGA	1.59	1.59	1.62	1.57
	GGG	0.72	0.72	0.7	0.71

Table S2. Analysis of simple sequence repeats (SSRs) in four Magnolia chloroplast genomes.

Score:W.S	Repeats(bp)	Start-End	original	Consensus patteern
56	15	62668-62703	62668	ATATACTTATAATAAG
			62683	ATATACTTATCATAAG
50	15	73025--73073	73025	AAATAGGTATAAAAT
			73040	AAATAGGTA-TAAA-AT

			73057	AAATAGGTATAAAAT
62	15	90517--	90517	TTCTTCCTATACCTA
		90547	90532	TTCTTCCTATACCTA
62	15	157340--	157340	ATAGGTATAGGAAGA
		157370	157355	ATAGGTATAGGAAGA
57	16	49068-	49068	TTTCTATT--ATTAGAAA
		49108	49086	TTT-TATTATTAGAAA
72	17	62703-	62703	ATTTCTAACAAGTACAA
		62738	62720	ATTTCTAACAAGTACAA
62	18	113155--	113155	TGTCATTGACATAAGAGA
		113220	113173	TGTCATTCA-ATCGAAGAAGA
			113193	T-G--TCATTGACATAAGAGA
78	18	132620--	132620	TATTGGTATTCTGATCAT
		132658	132638	TATTGGTATTCTGATCAT
62	18	132625--	132625	GTATTCTGATCATTATTG
		132664	132643	GTATTCTGATCATTATCA
62	18	134667--	134667	AATGACATCTCTTATGTC
		134732		
80	20	50685-	50685	AGTCTAGACCATGATACATA
		50724	50705	AGTCTAGACCATGATACATA
72	21	71884--	71884	TAAATCCAAGCGATCT-TTTCG
		71927	71905	TAAATCCAAGCGATCTTTTCG
72	21	87208--	87208	TTTTTCTTCTCTCTCTCTCTC
		87252	87252	TTTTTTTTCTCTCTCTCTCTC
126	21	92780--	92780	TTTGTCCAAGTCACTTC---TCTT
		92868	92804	TTTGTCCAAGTCACTTCTCTT
			92825	TTTGTCCAAGTCACTTCTCTT
			92846	TTTGTCTAAGTCACTTC-CTT
126	21	155019--	155019	AAAAAG-GAAGTGACTTAGAC
		155107	155039	AAAAAGAGAAGTGACTTGGAC
			155060	AAAAAGAGAAGTGACTTGGAC
			155081	AAAAAG--A-GAAGTGACTTAGAC
80	24	83188--	83188	TACGCCCACTCCTACGTGAACCAA
		83236	83212	TACGCCCATTCCTACGTGAACCAA
114	24	95234--	95234	TGACGATATCGATATTGATGATAG
		95290	95258	TGACGATATCGATATTGATGATAG
			134685	AATG--A-CATC-TCTT--ATGTC
			134708	AATGACATCTCTTATGTC
114	24	152597--	152597	ATATCGTCACTATCATCAATATCG
		152653	152621	ATATCGTCACTATCATCAATATCG

Score:M.C	Repeats(bp)	Start-End	original	Consensus patteern
59	12	9423--9465	9423	TTCTTTTCTTTA
			9435	TTCTTTTCTTTA

			9447	---TTCTTTTCTTTA
81	15	9412--9462	9412	TTTCTTTATCTTTCT
			9427	TTTCTTTA---TTCT
			9439	TTTCTTTATCTTTCT
62	15	6420--6450	6420	ATTACCAATACTGGA
			6450	ATTACCAATACTGGA
55	15	49051--49081	49051	ATTAGAAATTT-TATT
			49066	ATTAGAAATTTCTATT
56	15	62649--62684	62649	ATATACTTATAATAA-
			62664	ATATACTTATCATAAG
50	15	73037--73085	73037	AAATAGGTATAAAAT
			73052	AAATAGGTAGTAAATGT
			73069	AAAT-GG-AT-AAAT
62	15	90506--90536	90506	TTCTTCCTATACCTA
			90521	TTCTTCCTATACCTA
62	15	157510--157540	157510	ATAGGTATAGGAAGA
			157525	ATAGGTATAGGAAGA
64	16	118764--118795	118764	TGTACGAAGAATAATT
			118780	TGTACGAAGAATAATT
74	18	67258--67294	67258	AATGAACTTATAAAAAAA
			67276	AATGAACTTATAAAAAAA
62	18	113187--113252	113187	TGTCATTGACATAAGAGA
			113205	TGTCATTCA-ATCGAAGAAGA
			113225	TGGTATCATTGACATAAGAGA
141	18	132702--132776	132702	TATTGGCATTCTGATCAT
			132720	TATTGGTATTCTGATCAT
			132738	TATTGGTATTCTGATCAT
			132756	TATTGGTATTCTGATCAT
62	18	134794--134859	134794	AATGACATCTCTTATGTC
			134812	AATGATACCATCTTCTTCGAT-TG
			134835	AATGACATCTCTTATGTC
80	20	50668--50707	50668	AGTCTAGACCATGATACATA
			50688	AGTCTAGACCATGATACATA
63	21	14142--14186	14142	TTTTTTTATTATTGATTTCTT
			14163	TTTTTTTATTAATTGACTTATT
84	21	22169--22210	22169	TTTTACACTTCTTACTATTAG
			22190	TTTTACACTTCTTACTATTAG
84	21	44954--44995	44954	TAATGAATTAAAAAAGAGAGT
			44975	TAATGAATTAAAAAAGAGAGT
72	21	71893--71936	71893	TAAATCCAAGCGA-CTCTTTCG
			71914	TAAATCCAAGCGATCTTTTCG
			83229	TACGCCCACTCTTACGTGAACCAA
126	21	92769--	92769	TTTGTCTAAGTCACTTCGTTTCTT

		92857	92793	TTTGTCCAAGTCACTTCTCTT
			92814	TTTGTCCAAGTCACTTCTCTT
			92835	TTTGTCTAAGTCACTTC-CTT
126	21	155189--155277	155189	AAAAAG-GAAGTGACTTAGAC
			155209	AAAAAGAGAAGTGACTTGGAC
			155230	AAAAAGAGAAGTGACTTGGAC
			155251	AAAAAGAAACGAAGTGACTTAGAC
80	24	83205--83253	83205	TACGCCCACTCTTACGTGAACCAA
114	24	95223--95279	95223	TGACGATATCGATATTGATGATAG
			95247	TGACGATATCGATATTGATGATAG
114	24	152767--152823	152767	ATATCGTCACTATCATCAATATCG
			152791	ATATCGTCACTATCATCAATATCG
108	27	9412--9465	9412	TTTCTTTATCTTTCTTTTCTTTATTCT
			9439	TTTCTTTATCTTTCTTTTCTTTATTCT
Score:M.F	Repeats(bp)	Start-End	original	Consensus patteern
50	5	9404--9453	Copynumber: 9	TTTTC<br>TTTATC<br>-TTTC<br>TTTTC<br>TTTATTC<br>TTTTC<br>TTTATC<br>-TTTC<br>TTTTC
59	12	9416--9458	9416	TTCTTTTCTTTA
			9428	TTCTTTTCTTTA
			9440	TCTTTCTTTTCTTTA
62	15	6420--6450	6420	ATTACCAATACTGGA
			6435	ATTACCAATACTGGA
83	15	9404--9455	9404	TTTTCTTTATCTTTC
			9419	TTTTCTTTA---TTC
			9431	TTTTCTTTATCTTTC
55	15	49141--49171	49141	ATTAGAAATTT-TATT
			49156	ATTAGAAATTTCTATT
56	15	62732--62767	62732	ATATACTTATAATAA-
			62747	ATATACTTATCATAAG
50	15	73098--73146	73098	AAATAGGTATAAAAT
			73113	AAATAGGTAGTAAATGT
			73130	AAAT-GG-AT-AAAT
62	15	90582--90612	90582	TTCTTCCTATACCTA
			90597	TTCTTCCTATACCTA
62	15	157615--	157615	ATAGGTATAGGAAGA

		157645	157630	ATAGGTATAGGAAGA
62	18	113284--113349	113284	TGTCATTGACATAAGAGA
			113302	TGTCATTCA-ATCGAAGAAGA
			113322	TGGTATCATTGACATAAGAGA
105	18	132804--132860	132804	TATTGGCATTCTGATCAT
			132822	TATTGGTATTCTGATCAT
			132840	TATTGGTATTCTGATCAT
62	18	134878--134943	134878	AATGACATCTCTTATGTC
			134896	AATGATACCATCTTCTTCGAT-TG
			134919	AATGACATCTCTTATGTC
80	20	50761--50800	50761	AGTCTAGACCATGATACATA
			50781	AGTCTAGACCATGATACATA
78	20	118617--118655	118617	TTTTTTTAGGAAGAAGTTTC
			118637	TTTTTTTAGGAAGAAGTTT
56	21	14137--14181	14137	TTTTTTTATTATTGATTTCTT
			14158	TTTTTTTATTAATTGACTT-AT
63	21	14137--14181	14137	TTTTTTTATTATTGATTTCTT
			14158	TTTTTTTATTAATTGACTTATT
84	21	45041--45082	45041	TAATGAATTAAAAAAGAGAGT
			45062	TAATGAATTAAAAAAGAGAGT
72	21	71955--71998	71955	TAAATCCAAGCGA-CTCTTTCG
			71976	TAAATCCAAGCGATCTTTTCG
75	21	87279--87320	87279	TTTTTCTTCTCTCTCTTTCTC
			87300	TTTTTTTTCTCTCTCTTTCTC
168	21	92845--92954	92845	TTTGTCTAAGTCACTTCGTTTCTT
			92869	TTTGTCCAAGTCACTTCTCTT
			92890	TTTGTCCAAGTCACTTCTCTT
			92911	TTTGTCCAAGTCACTTCTCTT
			92932	TTTGTCTAAGTCACTTC-CTT
168	21	155273--155382	155273	AAAAAG-GAAGTGACTTAGAC
			155293	AAAAAGAGAAGTGACTTGGAC
			155314	AAAAAGAGAAGTGACTTGGAC
			155335	AAAAAGAGAAGTGACTTGGAC
			155356	AAAAAGAAACGAAGTGACTTAGAC
61	22	9406--9455	9406	TTCTTTATCTTTCTTTTCTTTA
			9428	TTCTTT-TCTTTATCTTTC-TT-
80	24	83260--83308	83260	TACGCCCACTCTTACGTGAACCAA
			83284	TACGCCCATTCCTACGTGAACCAA
114	24	95320--95376	95320	TGACGATATCGATATTGATGATAG
			95344	TGACGATATCGATATTGATGATAG
114	24	152851--152907	152851	ATATCGTCACTATCATCAATATCG
			152875	ATATCGTCACTATCATCAATATCG
110	27	9404--9458	9404	TTTTCTTTATCTTTCTTTTCTTTATTC

			9459	TTTTCTTTATCTTTCTTTTCTTTATTC
Score:M.M	Repeats(bp)	Start-End	original	Consensus patteern
59	12	9404--9446	9404	TTCTTTTCTTTA
			9416	TTCTTTTCTTTA
			9428	TCTTTCTTTTCTTTA
62	15	6408--6438	6408	ATTACCAATACTGGA
			6423	ATTACCAATACTGGA
81	15	9393--9443	9393	TTTCTTTATCTTTCT
			9408	TTTCTTTA---TTCT
			9420	TTTCTTTATCTTTCT
56	15	62747--62782	62747	ATATACTTATAATAA-
			62762	ATATACTTATCATAAG
50	15	73135--73183	73135	AAATAGGTATAAAAT
			73150	AAATAGGTAGTAAATGT
			73167	AAAT-GG-AT-AAAT
62	15	90643--90673	90643	TTCTTCCTATACCTA
			90658	TTCTTCCTATACCTA
62	15	157629--157659	157629	ATAGGTATAGGAAGA
			157644	ATAGGTATAGGAAGA
55	16	49156--49186	49146	ATTAGAAATTT-TATT
			49171	ATTAGAAATTTCTATT
63	17	62782--62817	62782	ATTTCTAACAAGTACAA
			62799	ATTTCTAATAAGTACAA
62	18	113318--113383	113318	TGTCATTGACATAAGAGA
			113336	TGTCATTCA-ATCGAAGAAGA
			113356	TGGTATCATTGACATAAGAGA
177	18	132809--132901	132809	TATTGGCATTCTGATCAT
			132827	TATTGGTATTCTGATCAT
			132845	TATTGGTATTCTGATCAT
			132863	TATTGGTATTCTGATCAT
			132881	TATTGGTATTCTGATCAT
62	18	134919--134984	134919	AATGACATCTCTTATGTC
			134937	AATGATACCATCTTCTTCGAT-TG
			134960	AATGACATCTCTTATGTC
64	19	67356--67395	67356	AATGAACTTATCAAAAAAA
			67375	AATGAACTTAT-AAAATAAA
80	20	50773--50812	50763	AGTCTAGACCATGATACATA
			50793	AGTCTAGACCATGATACATA
78	20	118626--118664	118626	TTTTTTTAGGAAGAAGTTTC
			118646	TTTTTTTAGGAAGAAGTTT
72	21	71993--72036	71993	TAAATCCAAGCGA-CTCTTTCG
			72014	TAAATCCAAGCGATCTTTTCG
63	21	14123--	14123	TTTTTTTATTATTGATTTCTT

		14167	14144	TTTTTTTATTAATTGACTTATT
84	21	22150--22191	22150	TTTTACACTTCTTACTATTAG
			22171	TTTTACACTTCTTACTATTAG
84	21	45061--45102	45061	TAATGAATTAAAAAAGAGAGT
			45082	TAATGAATTAAAAAAGAGAGT
75	21	87340--87381	87340	TTTTTCTTCTCTCTCTTTCTC
			87361	TTTTTTTTCTCTCTCTTTCTC
126	21	92906--92994	92906	TTTGTCTAAGTCACTTCGTTTCTT
			92930	TTTGTCCAAGTCACTTCTCTT
			92951	TTTGTCCAAGTCACTTCTCTT
			92972	TTTGTCTAAGTCACTTC-CTT
126	21	155308--155396	155308	AAAAAG-GAAGTGACTTAGAC
			155328	AAAAAGAGAAGTGACTTGGAC
			155349	AAAAAGAGAAGTGACTTGGAC
			155370	AAAAAGAAACGAAGTGACTTAGAC
88	22	81813--81856	81813	AATTTCTTCTTTTCTTTCTTTT
			81835	AATTTCTTCTTTTCTTTCTTTT
80	24	83322--83370	83322	TACGCCCACTCTTACGTGAACCAA
			83346	TACGCCCATTCCTACGTGAACCAA
114	24	95360--95416	95360	TGACGATATCGATATTGATGATAG
			95384	TGACGATATCGATATTGATGATAG
114	24	152886--152942	152886	ATATCGTCACTATCATCAATATCG
			152910	ATATCGTCACTATCATCAATATCG
108	27	9393-9446	9393	TTTCTTTATCTTTCTTTTCTTTATTCT
			9420	TTTCTTTATCTTTCTTTTCTTTATTCT

Table S3. Length of exons and introns in genes with introns in the Magnolia chloroplast genome.

Species	Gene	Location	ExonI(bp)	IntronI(bp)	ExonII(bp)	IntronII(bp)	Exon III(bp)
W.S	*trnK-UUU*	*LSC*	37	2488	35		
	rps16	*LSC*	41	825	253		
	trnS-CGA	*LSC*	32	749	61		
	atpF	*LSC*	145	706	410		
	rpoC1	*LSC*	432	734	1614		
	ycf3	*LSC*	124	733	232	731	151
	trnL-UAA	*LSC*	35	484	50		
	trnC-ACA	*LSC*	39	565	56		
	clpP	*LSC*	71	783	291	630	244
	petB	*LSC*	6	784	642		
	rpl2	*IRb*	385	661	431		
	ndhB	*IRb*	775	700	758		
	trnE-UUC	*IRb*	32	933	40		
	trnA-UGC	*IRb*	37	799	36		
	ndhA	*SSC*	553	1070	539		
	trnA-UGC	*IRa*	37	799	36		
	trnE-UUC	*IRa*	32	933	40		

	ndhB	*IRa*	775	700	758		
	rpl2	*IRa*	385	661	431		
M.C	*trnK-UUU*	*LSC*	37	2491	35		
	rps16	*LSC*	41	823	248		
	trnS-CGA	*LSC*	32	748	61		
	atpF	*LSC*	145	707	410		
	rpoC1	*LSC*	432	734	1635		
	ycf3	*LSC*	124	740	232	729	151
	trnS-UGA	*LSC*	36	54	33		
	trnL-UAA	*LSC*	35	491	50		
	trnC-ACA	*LSC*	39	565	56		
	clpP	*LSC*	71	783	291	630	244
	petB	*LSC*	6	784	642		
	rpl2	*IRb*	385	661	431		
	ndhB	*IRb*	775	700	758		
	trnE-UUC	*IRb*	32	941	40		
	trnA-UGC	*IRb*	37	799	36		
	ndhA	*SSC*	553	1077	539		
	ycf1	*SSC*	1941	36	3600		
	trnA-UGC	*IRa*	37	799	36		
	trnE-UUC	*IRa*	32	941	40		
	ndhB	*IRa*	775	700	758		
	rpl2	*IRa*	385	661	431		
M.F	*trnK-UUU*	*LSC*	37	2490	35		
	rps16	*LSC*	41	826	248		
	trnS-CGA	*LSC*	32	752	61		
	atpF	*LSC*	145	707	410		
	rpoC1	*LSC*	432	734	1614		
	ycf3	*LSC*	124	740	232	730	151
	trnL-UAA	*LSC*	35	491	50		
	trnC-ACA	*LSC*	39	565	56		
	clpP	*LSC*	71	781	291	631	244
	petB	*LSC*	6	784	642		
	rpl2	*IRb*	385	661	431		
	ndhB	*IRb*	775	700	758		
	trnE-UUC	*IRb*	32	941	40		
	trnA-UGC	*IRb*	37	799	36		
	ndhA	*SSC*	553	1078	539		
	trnA-UGC	*IRa*	37	799	36		
	trnE-UUC	*IRa*	32	941	40		
	ndhB	*IRa*	775	700	758		
	rpl2	*IRa*	385	661	431		
M.M	*trnK-UUU*	*LSC*	37	2490	35		
	rps16	*LSC*	41	813	241		
	trnS-CGA	*LSC*	32	751	61		
	atpF	*LSC*	145	706	410		
	rpoC1	*LSC*	432	734	1635		
	ycf3	*LSC*	124	737	232	730	151
	trnL-UAA	*LSC*	35	491	50		
	trnC-ACA	*LSC*	39	565	56		
	clpP	*LSC*	71	782	291	629	244
	petB	*LSC*	6	784	642		
	rpl2	*IRb*	385	661	431		

ndhB	*IRb*	775	700	758
trnE-UUC	*IRb*	32	941	40
trnA-UGC	*IRb*	37	799	36
NdhA	*SSC*	553	1075	539
ycf1	*SSC*	1941	54	3600
trnA-UGC	*IRa*	37	799	36
trnE-UUC	*IRa*	32	941	40
ndhB	*IRa*	775	700	758
rpl2	*IRa*	385	661	431

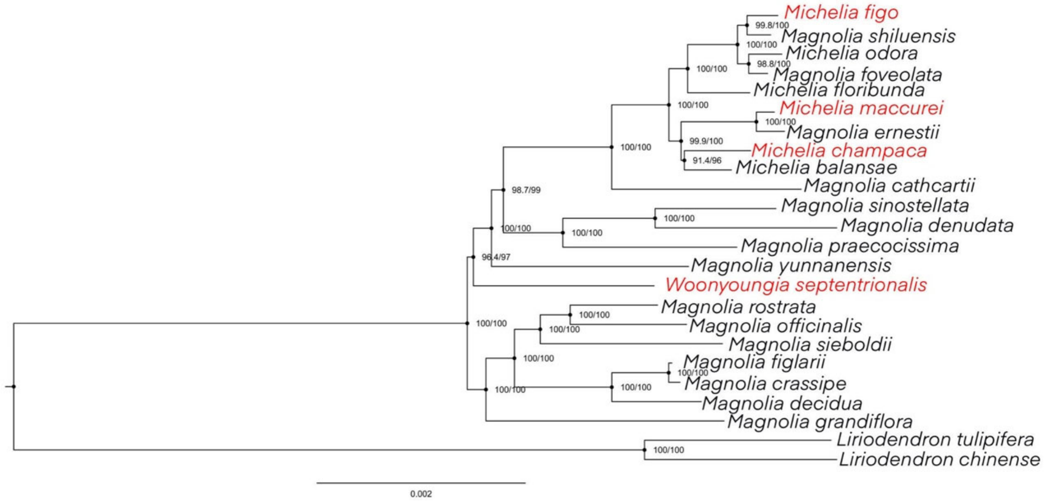

Figure S1. Phylogenetic analysis of 24 Magnoliaceae species based on Maximum Likelihood(ML) inference.

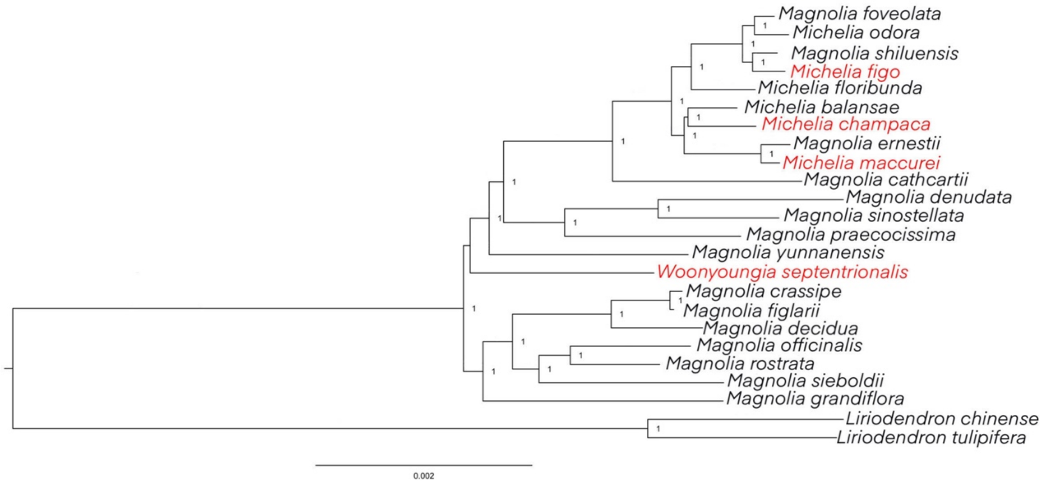

Figure S2. Phylogenetic analysis of 24 magnolia species obtained from new Bayesian Inference(BI) analysis.
